# Modified exosomes for targeted delivery of doxorubicin and carvedilol to mitochondria in breast cancer cells

**DOI:** 10.1038/s41598-025-22156-2

**Published:** 2025-11-03

**Authors:** Tooba Soudi, Fatemeh Bagheri, Seyed Abbas Shojaosadati, Mohsen Rezaei, Ehsan Motamedian

**Affiliations:** 1 Department of Biotechnology, Faculty of Chemical Engineering, Tarbiat Modares University, Tehran, Iran; 2 Department of Toxicology, Faculty of Medical Sciences, Tarbiat Modares University, Tehran, Iran

**Keywords:** Drug repurposing, Carvedilol, Doxorubicin, Breast cancer, Exosomal drug delivery, Dual-drug delivery, Breast cancer, Cancer therapy

## Abstract

Carvedilol (CAR), a well-established β-blocker and a promising pharmacological agent for mitigating doxorubicin (DOX)-induced cardiotoxicity, has also demonstrated anticancer effects against breast cancer cells. Given the seemingly contradictory effects of CAR and DOX treatment on cardiomyocytes and cancer cells, particularly regarding mitochondrial function, this study investigates the synergistic potential of DOX and CAR through various delivery systems, including free form, exosomal formulations, and mitochondria-targeted delivery systems. The Chou-Talalay analysis demonstrated that combining DOX with CAR at a 1:1 ratio reduced DOX’s IC50 in MCF-7 (from 0.274 µM to 0.191 µM) and MDA-MB-231 (from 0.138 µM to 0.086 µM) cells. Drug loading in exosomal formulations was achieved with approximately 24% efficiency, maintaining the optimum ratio. While CAR exhibited minimal cytotoxicity at concentrations below 2 µM, its combination with DOX enhanced anticancer effects. Mitochondrial-targeted delivery (TPP-Exo-DOX-CAR) showed reduced cytotoxicity compared to TPP-Exo-DOX formulation. ROS production analysis and mitochondrial membrane potential assessments revealed that CAR’s protective effects become dominant in mitochondria-targeted delivery in vitro. Migration studies demonstrated significant inhibition of cell motility in CAR containing formulations. These findings suggest that while the DOX and CAR combination shows promise in cancer treatment, the delivery system significantly impacts their therapeutic efficacy, particularly in mitochondrial-targeted applications.

**Supplementary Information:**

The online version contains supplementary material available at 10.1038/s41598-025-22156-2.

## Introduction

 Cancer remains a significant global health challenge, with an estimated 19–20 million new cancer cases diagnosed annually. Breast cancer is the most common cancer among women and accounts for a substantial proportion of cancer-related mortality worldwide^1^. Despite chemotherapy being a cornerstone of treatment for advanced malignancies, its clinical application is substantially hindered by numerous limitations. These challenges include severe systemic side effects, inadequate drug bioavailability, high dosage requirements, narrow therapeutic windows, the emergence of multidrug resistance, and a lack of precise cancer cell targeting. The continuous development of innovative treatment strategies is paramount to improving patient outcomes and reducing therapeutic limitations.

Doxorubicin (DOX), a potent anthracycline chemotherapeutic agent, has long been an important drug in treating various solid tumors and leukemia. Despite its remarkable antitumor efficacy, primarily mediated through topoisomerase II inhibition and DNA damage induction; DOX is critically constrained by severe systemic side effects. A major limitation of DOX is its significant cardiotoxicity, which characterized by mechanisms including reactive oxygen species (ROS) generation, oxidative stress, increased intracellular iron levels, and mitochondrial dysfunction. These adverse effects can lead to profound cardiac complications such as arrhythmia, cardiomyopathy, and potentially congestive heart failure, substantially limiting the drug’s long-term therapeutic potential^2^.

Carvedilol (CAR) has emerged as a promising pharmacological agent in mitigating DOX-induced cardiotoxicity, originally approved for hypertension and heart failure management. This non-selective β-blocker demonstrates remarkable properties, including potent antioxidant, anti-inflammatory, and mitochondrial protective effects. Its unique cardioprotective mechanisms involve preventing ROS production, chelating free iron, maintaining mitochondrial membrane potential (MMP), and preserving cellular energetic homeostasis^3,4^. Critically, CAR has been shown to counteract DOX’s cardiac toxicity without compromising the drug’s anticancer efficacy^5,6^.

Intriguingly, CAR has demonstrated independent anticancer properties that extend beyond its cardioprotective role. Research has revealed its potential to inhibit cancer cell proliferation, induce apoptosis^7–9^, and reverse multidrug resistance (MDR) by inhibiting P-glycoprotein, an ATP binding cassette transporter mediating drug efflux in cancer cells. CAR can enhance the cytotoxicity of various chemotherapeutic agents at clinically achievable concentrations^10^.

The drug’s interaction with mitochondria presents a complex narrative. While CAR is known for its mitochondrial protective properties in non-cancerous cells, emerging evidence suggests it can induce mitochondrial damage in specific cancer cell lines. Studies on C6 glioma spheroids revealed significant mitochondrial alterations, including swelling, cristae damage, and intramitochondrial myelin figure formation. This finding contrasts with its previously established cardioprotective mechanisms^8^.

These seemingly contradictory observations raise critical questions about CAR’s potential role in cancer treatment. Specifically, could CAR enhance DOX’s anticancer effects, potentially creating a synergistic combination? Given that both drugs significantly impact mitochondrial function, an intriguing possibility emerges: might their strategic targeting of mitochondria amplify their overall anticancer efficacy?

To address these questions, we designed an innovative exosome-based drug delivery system. Exosomes (Exo), nanoscale extracellular vesicles ranging from 30 to 100 nm, offer exceptional promise in targeted cancer therapeutics. Their inherent biocompatibility, low immunogenicity, and ability to encapsulate diverse therapeutic payloads make them ideal nanocarriers^11,12^. Particularly compelling are tumor cell-derived exosomes, which possess unique surface proteins facilitating selective cancer cell binding and uptake^13,14^.

The electrochemical gradient generated by proton pumps (Complexes I, III, and IV) results in a strong negative membrane potential across the inner mitochondrial membrane (−150 to −180 mV). This potential is remarkably greater than the plasma membrane potential (−60 to −90 mV), which ultimately enables selective mitochondria targeting of mitochondria by lipophilic cations^15,16^. Mitochondrial targeting represents a sophisticated approach to enhancing drug delivery. The delocalized lipophilic cations like triphenylphosphonium (TPP) could be used to achieve remarkable mitochondrial accumulation. TPP derivatives, characterized by their positively charged phosphorus atom surrounded by lipophilic phenyl groups, demonstrate a 1,000-fold higher mitochondrial accumulation than cytosolic concentrations^17^.

This study comprehensively investigates the synergistic potential of DOX and CAR combinations across three distinct delivery modalities: free drug formulations, exosome-loaded preparations, and mitochondria-targeted delivery systems. The Drug combination effects were analyzed by the Chou-Talalay method. Drug interactions were evaluated through assessing mitochondrial membrane depolarization, reactive oxygen species generation, apoptosis induction, and cellular migration capacity on two breast cancer cell lines: MDA-MB-231 and MCF-7. This integrated approach elucidates the mechanistic basis of drug synergy while exploring novel therapeutic strategies for enhanced breast cancer treatment efficacy.

## Results

### Combination effects of DOX and CAR

Plasma concentrations of DOX vary from low ng/mL to µg/mL levels, primarily depending on treatment regimens and administration protocols. Clinical studies have established that DOX concentrations in human blood typically range between 0.025 and 0.250 µM^18,19^. CAR’s pharmacokinetic properties, characterized by rapid absorption and peak plasma concentrations between 0.01 and 0.46 µM, typically achieved within 1–2 h post-administration^20^. Based on these concentrations, two clinically relevant molar ratios of DOX to CAR (2:1 and 1:1) were selected to investigate potential synergistic interactions. The combination effects were evaluated using the Chou-Talalay method in two breast cancer cell lines: MCF-7 and MDA-MB-231. Single agent cytotoxicity studies (provided in Supplementary Fig. S1) revealed that CAR exhibited minimal cytotoxic effects (< 5% in MDA-MB-231 and < 10% in MCF-7) at concentrations below 2 µM following 48 h exposure. In comparison DOX exerted cell inhibition effects for more than 80% in both cell lines in these concentrations.

CompuSyn software was employed to analyze dose-effect relationships for the drug combinations (Supplementary Fig. S2). Although CAR demonstrated significantly lower toxicity than DOX as a single agent, the dose-response curves revealed that combination therapy exhibited toxicity levels comparable to DOX monotherapy. Furthermore, reducing the DOX proportion from 2:1 to 1:1 (DOX: CAR) did not substantially decrease the combination’s toxicity, as evidenced by the nearly overlapping dose-response curves. Quantitative analysis of drug interactions at Fa = 0.5 revealed significant reductions in IC50 of DOX in combination with CAR. The IC50 values provided in Table 1 were calculated by CompuSyn software, while statistical comparison of dose-response curves was performed using GraphPad Prism software. In MCF-7 cells, the IC50 of DOX decreased from 0.274 µM as a single agent to 0.243 µM and 0.191 µM at 2:1 and 1:1 DOX: CAR ratios (P value < 0.001), respectively. Also, in MDA-MB-231 cells, the IC50 decreased from 0.138 µM to 0.097 µM and 0.086 µM under the same conditions (P value < 0.05). As given in Table 1, CAR had IC50 values of 103.01 µM and 118.34 µM for MCF-7 and MDA-MB-231 cell lines.

**Table 1:** IC50 and CI values of DOX, CAR, and their combinations at Fa = 0.5.

IC50 Doses (Fa = 0.5)	MCF-7			MDA-MB-231		
Drug/Combo	Dose DOX (µM)	Dose of CAR (µM)	CI	Dose of DOX (µM)	Dose of CAR (µM)	CI
DOX	0.274	-	-	0.138	-	-
CAR	-	103.01	-	-	118.34	-
DOX-CAR 1:1	0.191	0.191	0.696	0.086	0.086	0.623
DOX-CAR 2:1	0.244	0.122	0.847	0.097	0.048	0.701

**Fig. 1:**
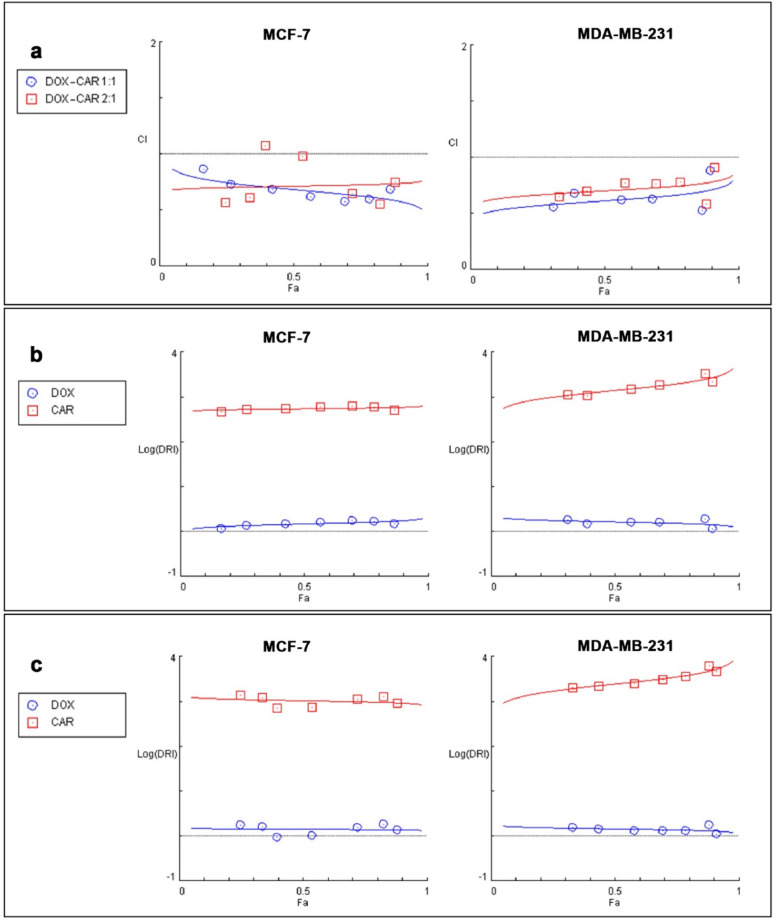
**Synergy assessment using CI and DRI indexes** The Chou-Talalay method was applied to evaluate drug synergy in MCF-7 and MDA-MB-231 cells. The CI-Dose plots (**a**) show the synergistic interaction (CI < 1) between DOX and CAR across different doses for 1:1 and 2:1 combination ratio, with lower CI values indicating stronger synergy. The logarithmic DRI plots for 1:1 (**b**) and 2:1 (**c**) drug ratios demonstrate the extent to which drug doses can be reduced while maintaining efficacy.

Combination index (CI) analysis revealed synergistic interactions (CI < 1) across all tested concentrations in both cell lines (Fig. 1). The 1:1 molar ratio exhibited higher synergistic effects compared to the 2:1 ratio, as evidenced by lower CI values. Dose Reduction Index (DRI) analysis demonstrated that CAR enhanced DOX cytotoxicity. Briefly, in MCF-7 cells, CAR increased DOX’s treatment efficacy by an average of 49.9% and 43.4% at 1:1 and 2:1 ratio, respectively. Similarly, in MDA-MB-231 cells, it enhanced DOX cytotoxicity by average values of 58.7% and 38.4% at these respective ratios. The detailed DRI values for each Fa are shown in Fig. 1. The 1:1 ratio showed the best CI and DRI for both cell lines; therefore, this ratio was selected for the following tests and will be referred as DOX-CAR in the rest of the text.

## Exosome isolation and drug loading

Exosomes obtained from the conditioned media of the MCF-7 and MDA-MB-231 cell lines were comprehensively characterized based on their size, surface charge, specific surface markers (CD63 and CD9), and structural morphology, as illustrated in Supplementary Fig. S3 and Fig. S4 for each cell line, respectively. DLS analysis of the isolated exosomes revealed a highly consistent and narrow size distribution, with over 80% of the particles falling within the 30–100 nm range. The mean particle diameters were 94.9 ± 21.9 nm for MCF-7-derived exosomes and 99.8 ± 18.06 nm for those derived from MDA-MB-231 cells. The surface charge of the exosomes was assessed through zeta potential measurements, yielding values of −36.7 ± 4.5 mV for MCF-7 exosomes and − 35.4 ± 3.1 mV for MDA-MB-231 exosomes. TEM was used to visualize the exosomes, which exhibited the characteristic spherical morphology typical of extracellular vesicles.

Further characterization of the exosomal surface markers CD63 and CD9 was performed using flow cytometry. These markers provided additional evidence for successfully isolating and characterizing of exosomes from the cell lines under study. These findings collectively validate the isolation procedure’s efficacy and confirm that the extracted extracellular vesicles are exosomes.

Drug loading was performed using the sonication method, which demonstrated higher loading efficiency compared to alternative methods such as incubation and freeze-thaw cycles^21^. Exo-DOX-CAR and TPP-Exo-DOX-CAR characterization is detailed in Table 2. The exosomes showed high drug loading capacity for DOX and CAR with a total loading efficiency of approximately 24%. Exosomes have a natural capacity for cargo loading due to their endogenous role in intercellular communication. The sonication process further enhances drug loading by temporarily disrupting the exosomal membrane, creating transient pores that facilitate drug diffusion into the vesicle interior. Upon cessation of sonication, the membrane rapidly reseals, effectively trapping the drug molecules inside. Their ability to encapsulate hydrophobic small molecules within the lipid bilayer and accommodate hydrophilic compounds within their aqueous core contributes to the observed high loading efficiency^21^. The flexible and dynamic nature of exosomal membrane may also play a role in stabilizing the loaded drug and enhancing retention. The drugs were introduced to the Exo suspension at a 1:1 molar ratio. The loading process achieved a near-equimolar ratio of DOX to CAR (1:0.93), approaching the intended 1:1 molar ratio. This slight deviation from the target ratio could be attributed to the differential incorporation tendencies of the two drugs into the exosomal membrane.

Drug loading via sonication led to exosomal size and surface charge alterations, while TPP conjugation further increased particle size (Table 2). The MCF-7 derived exosomes increased in size from 94.9 ± 21.7 nm to 142.9 ± 10.0 nm for Exo-DOX-CAR and 157.1 ± 13.5 nm for TPP-Exo-DOX-CAR. Similarly, MDA-MB-231 exosomes demonstrated a size increase from 99.8 ± 18.1 nm to 142.7 ± 11.3 nm and 156.2 ± 7.0 nm, respectively. The zeta potential increased to −23.3 ± 4.9 mV and − 21.5 ± 6.5 mV for MCF-7 and MDA-MB-231 derived exosomes, respectively. The notable shift in zeta potential from negative to positive following TPP conjugation validates successful TPP conjugation to the Exo-DOX-CAR surface. The changes in zeta potential distribution among Exo, Exo-DOX-CAR, and TPP-Exo-DOX-CAR are illustrated in Supplementary Fig. S5.

**Table 2:** Characterization of drug loaded and TPP conjugated Exo.

Cell line	Sample	Size (nm)	Zeta Potential (mV)	Loading Capacity (%)			DOX/CAR Molar ratio
				DOX	CAR	Total	
MCF-7	Exo-DOX-CAR	142.9 ± 10.0	−23.3 ± 4.9	14.2 ± 2.8	9.8 ± 1.6	24.0 ± 4.4	1:0.92
	TPP-Exo-DOX-CAR	157.1 ± 13.5	14.2 ± 0.8	13.9 ± 1.7	9.7 ± 1.0	23.6 ± 2.7	1:0.93
MDA-MB-231	Exo-DOX-CAR	142.7 ± 11.3	−21.5 ± 6.4	14.9 ± 2.7	10.1 ± 1.9	25.0 ± 4.6	1:0.91
	TPP-Exo-DOX-CAR	156.2 ± 7.0	13.4 ± 0.9	14.7 ± 2.3	10.2 ± 1.3	24.9 ± 3.6	1:0.93

The DOX and CAR release profiles from Exo-DOX-CAR were assessed under conditions simulating both physiological (pH 7.4) and endolysosomal (pH 5.5) environments. At pH 5.5, which mimics the acidic environment of endolysosomes, approximately 60% of DOX was released over 48 h, whereas only 40% was released under physiological pH conditions (pH 7.4). CAR, in comparison, demonstrated even greater pH sensitivity. This enhanced release of CAR is due to its low intrinsic water solubility, which increases markedly under acidic conditions. Specifically, CAR’s solubility is observed to increase threefold under acidic pH (from 0.013 mg/mL to 0.037 mg/mL^22^), leading to a release rate that is twice as high at pH 5.5 compared to pH 7.4. The release profiles are shown in Supplementary Fig. S6. The release profile from nanoparticles plays a crucial role in their overall therapeutic performance. Various strategies have been employed to modify bio-based nanoparticles to enhance their delivery efficiency^23^. Notably, exosomes, as natural carriers, demonstrated pH-sensitive drug release behavior.

## Intracellular distribution of Exosomal formulations

**Fig. 2:**
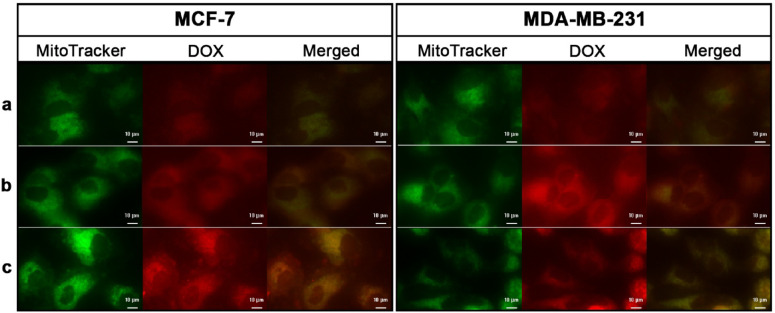
**Intracellular distribution of DOX in free form and exosomal formulations** Intracellular distribution of free DOX (**a**), Exo-DOX (**b**), and TPP-Exo-DOX (**c**) after 8 h incubation with MCF-7 and MDA-MB-231 cells. Green fluorescence indicates mitochondria stained with MitoTracker Green, red fluorescence represents DOX emission, and yellow spots in the merged images demonstrate co-localization. The enhanced yellow signal in TPP-Exo-DOX treated cells confirms successful mitochondrial targeting of the TPP-conjugated formulation.

MCF-7 and MDA-MB-231 cells were treated with Free DOX, Exo-DOX, and TPP-Exo-DOX, and their subcellular localization was analyzed using a Cell Imaging Multi-Mode Reader. In Fig. 2, mitochondria were stained with Mitotracker and visualized in green, and the DOX fluorescence was shown in red. Free DOX displayed the lowest fluorescence intensity, indicating minimal cellular uptake compared to Exo-DOX and TPP-Exo-DOX. The exosomal formulations significantly enhanced DOX uptake. In cells treated with Exo-DOX, red fluorescence was distributed throughout the cytoplasm, suggesting cellular uptake without specific targeting. Free DOX and Exo-DOX showed negligible yellow fluorescence in the merged image. In contrast, cells treated with TPP-Exo-DOX displayed red fluorescence as distinct spots colocalized with green fluorescence, indicating selective mitochondrial accumulation of the TPP-conjugated formulation. TPP-Exo-DOX demonstrated significant mitochondrial accumulation, as evidenced by prominent yellow fluorescence.

## Cytotoxicity and antitumor effects

**Fig. 3:**
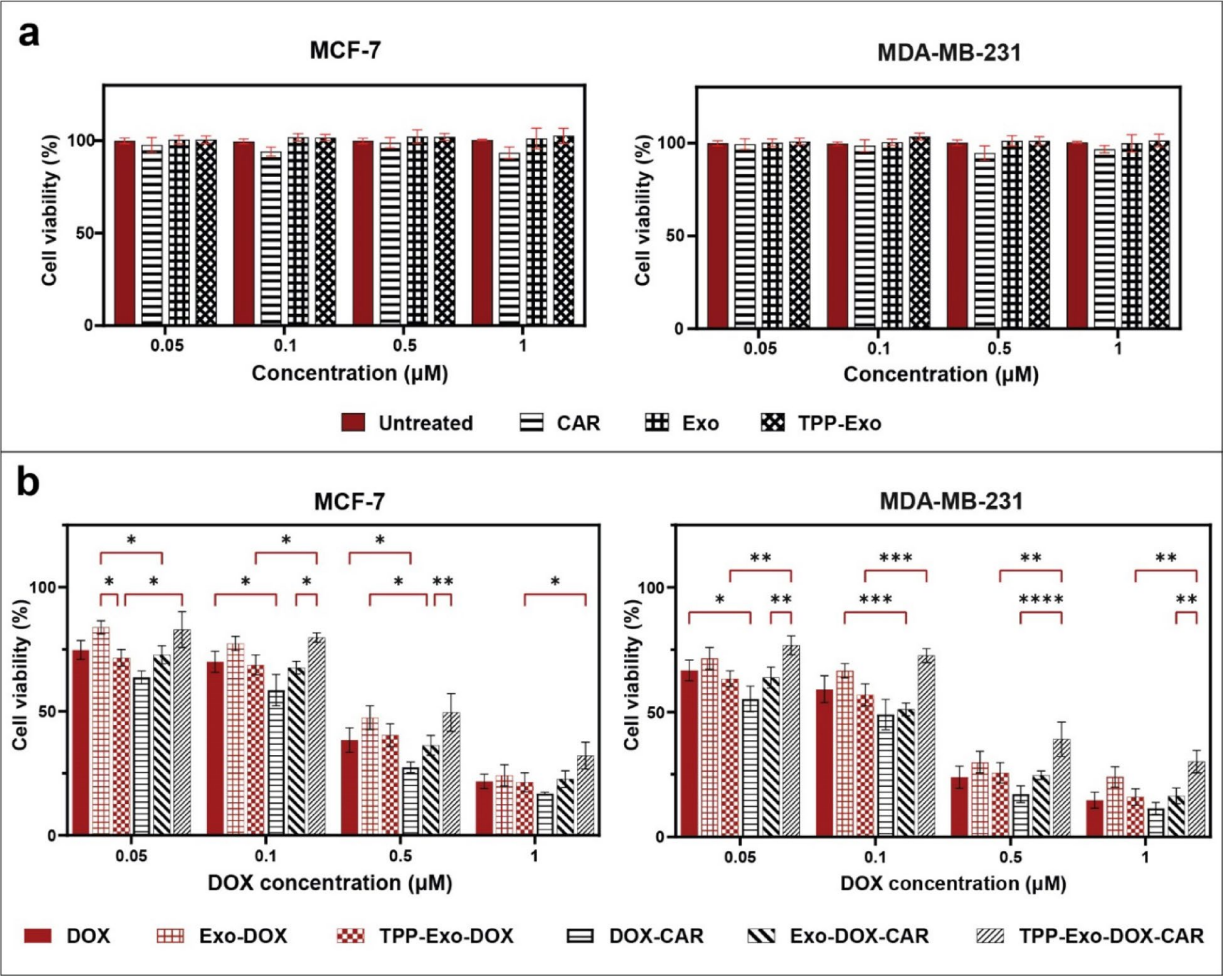
**Cell viability of MCF-7 and MDA-MB-231 cells after 48 h treatment** (**a**) Cell viability after treatment with blank carriers and CAR alone. (**b**) Cell viability of various DOX containing formulations. Results are shown as mean ± SD (*n* = 3). Statistical significance is indicated by: *P < 0.05, **P < 0.01, ***P < 0.001, ****P < 0.0001.

The cytotoxicity of blank exosomes was shown in Fig. 3a; neither Exo and TPP-Exo nor free CAR showed significant proliferation or toxicity compared to control in tested concentrations (Fig. 3a). Since CAR had negligible cytotoxicity in comparison with DOX, all formulations were compared by their DOX content, the CAR toxicity is also shown as control in Fig. 3a. The Cytotoxicity of DOX in free form and Exo-DOX, TPP-Exo-DOX, free DOX-CAR, Exo-DOX-CAR, TPP-Exo-DOX-CAR formulations on MCF-7 and MDA-MB-231 cells was examined after 48 h treatment using MTT assay as shown in Fig. 3b. In MCF-7 cells, free DOX showed an IC50 of 0.274 µM, while the Exo-DOX had a slightly higher IC50 of 0.350 µM. The TPP-Exo-DOX demonstrated improved potency with an IC50 of 0.225 µM. Combined with CAR, free DOX-CAR exhibited an IC50 of 0.191 µM, and Exo-DOX-CAR maintained similar effectiveness with an IC50 at 0.218 µM. However, TPP-Exo-DOX-CAR showed reduced potency with an IC50 of 0.444 µM.

In the MDA-MB-231 cell line, Free DOX and Exo-DOX showed an IC50 of 0.138 µM and 0.196 µM, respectively. The TPP-Exo-DOX demonstrated the IC50 at 0.120 µM. The CAR combinations followed a similar pattern to MCF-7 cells: free DOX-CAR showed the lowest IC50 at 0.086 µM, Exo-DOX-CAR required 0.108 µM, and TPP-Exo-DOX-CAR was less effective with IC50 at 0.304 µM.

Although a reduction in toxicity in Exo-DOX or Exo-DOX-CAR compared to the free drugs are observed in Fig. 3b, the differences compared to free drugs were not significant in both cell lines. The differences between DOX and Exo-DOX or DOX-CAR and Exo-DOX-CAR were not significant, which shows that exosome loading had a minor effect on DOX toxicity in vitro in both cell lines. Consistent with DOX-CAR, which is more toxic than DOX, the exosomal formulation of Exo-DOX-CAR was more cytotoxic than Exo-DOX; therefore exosome formulations effectively preserved drug toxicity well in the single drug delivery and dual drug delivery modes.

Mitochondrial targeting of Exo-DOX by TPP conjugation also increased Exo-DOX’s toxicity. As observed by fluorescent microscopic images, TPP conjugation could increase cell entrance and DOX accumulation in mitochondria (Fig. 2). Previous researches also confirm that mitochondrial targeting of DOX could increase DOX toxicity and also has positive effect on inhibition of drug resistance^24,25^. Several studies show mitochondrial targeting of DOX could increase its toxicity by targeting mitochondrial DNA or by interfering in mitochondrial respiration and increasing ROS production and triggering Apoptosis^26^.

In contrast to TPP-Exo-DOX, mitochondrial targeting of DOX-CAR (TPP-Exo-DOX-CAR) reduced DOX-CAR toxicity compared to free DOX-CAR or Exo-DOX-CAR formulations and also reduced cytotoxicity compared to TPP-Exo-DOX. It seems that CAR neutralized the enhanced toxicity of DOX, which resulted from the mitochondrial targeting of DOX.

Mitochondrial toxicity induction is closely related to ROS production and mitochondrial dysfunction. Therefore, ROS production and MMP were assessed to elucidate the underlying mechanism for CAR and DOX interactions.

## Intracellular ROS production

**Fig. 4:**
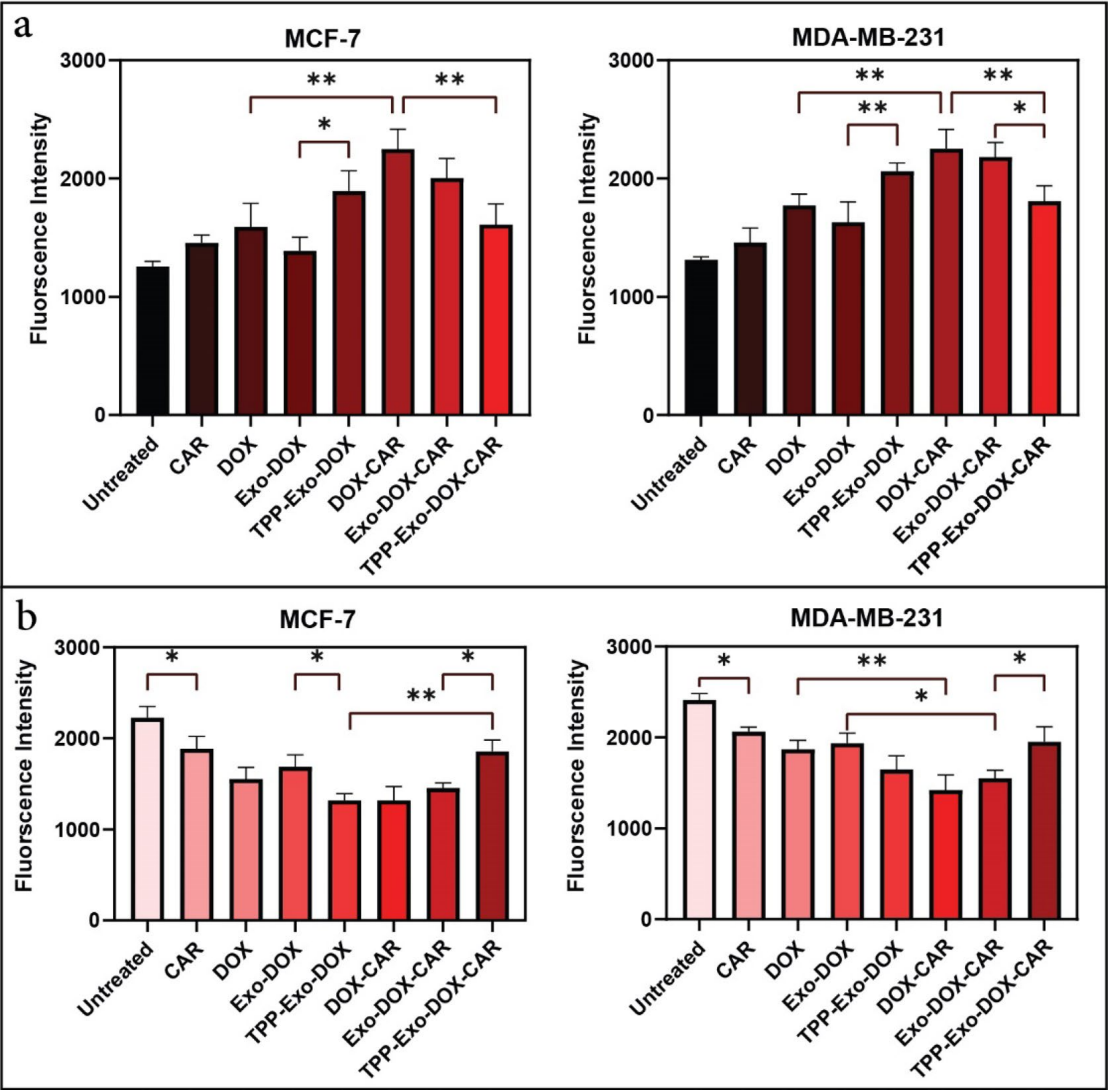
**Effects of different formulations on ROS production and MMP in cell lines MCF-7 and MDA-MB-231** (**a**) ROS level is measured by DCF fluorescence intensity, where increased fluorescence indicates elevated ROS production. (**b**) Mitochondrial function is assessed by Rh123 fluorescence, where decreased fluorescence represents mitochondrial depolarization. CAR induced ROS production and mitochondrial dysfunction when delivered as free DOX-CAR and Exo-DOX-CAR, while the TPP-Exo-DOX-CAR formulation demonstrated protective effects on mitochondrial integrity. Results are shown as mean ± SD (*n* = 3). Statistical significance is indicated by: *P < 0.05, **P < 0.01.

ROS content was evaluated using DCF fluorescence measurement via both microplate reader and flow cytometry. Both methods demonstrated consistent results. DOX concentration was set to 0.1 µM in all DOX-containing formulations, with DOX-CAR formulations additionally containing 0.1 µM CAR. Results are presented in Fig. 4a. The data, as mentioned earlier, revealed that CAR showed no significant toxicity at this concentration; however, CAR slightly increased ROS levels compared to untreated cells. Treatment with DOX-CAR led to significantly elevated ROS levels compared to DOX alone. In MCF-7 cells, the enhanced DCF fluorescence intensity revealed a 2.0-fold increase in ROS generation following DOX-CAR treatment. In MDA-MB-231 cells, DOX-CAR treatment resulted in a 2.9-fold increase in ROS content relative to DOX treatment.

TPP-Exo-DOX demonstrated higher ROS content compared to Exo-DOX. However, TPP-Exo-DOX-CAR induced lower ROS content than TPP-Exo-DOX or Exo-DOX-CAR, suggesting that mitochondrial targeting of the DOX-CAR combination reduced its toxicity. ROS analysis by flow cytometry (Fig. S7) confirmed these findings.

### Mitochondrial membrane depolarization

The MMP serves as a critical indicator of cellular viability. Rh123 was used to assess MMP in MCF-7 and MDA-MB-231 cell lines after 24 h exposure to 0.1 µM DOX (with 0.1 µM CAR in DOX-CAR formulations). Rh123 fluorescence undergoes quenching during mitochondrial energization, with the fluorescence decay rate directly proportional to the membrane potential. Figure 4b illustrates the Rh123 fluorescence intensity.

The results revealed that CAR significantly disrupted MMP in both MCF-7 and MDA-MB-231 cell lines. The fluorescence intensity showed significant MMP loss in DOX-CAR and Exo-DOX-CAR groups compared to DOX and Exo-DOX groups, indicating that DOX-CAR induced greater mitochondrial damage than DOX.

## Apoptotic cell death

**Fig. 5:**
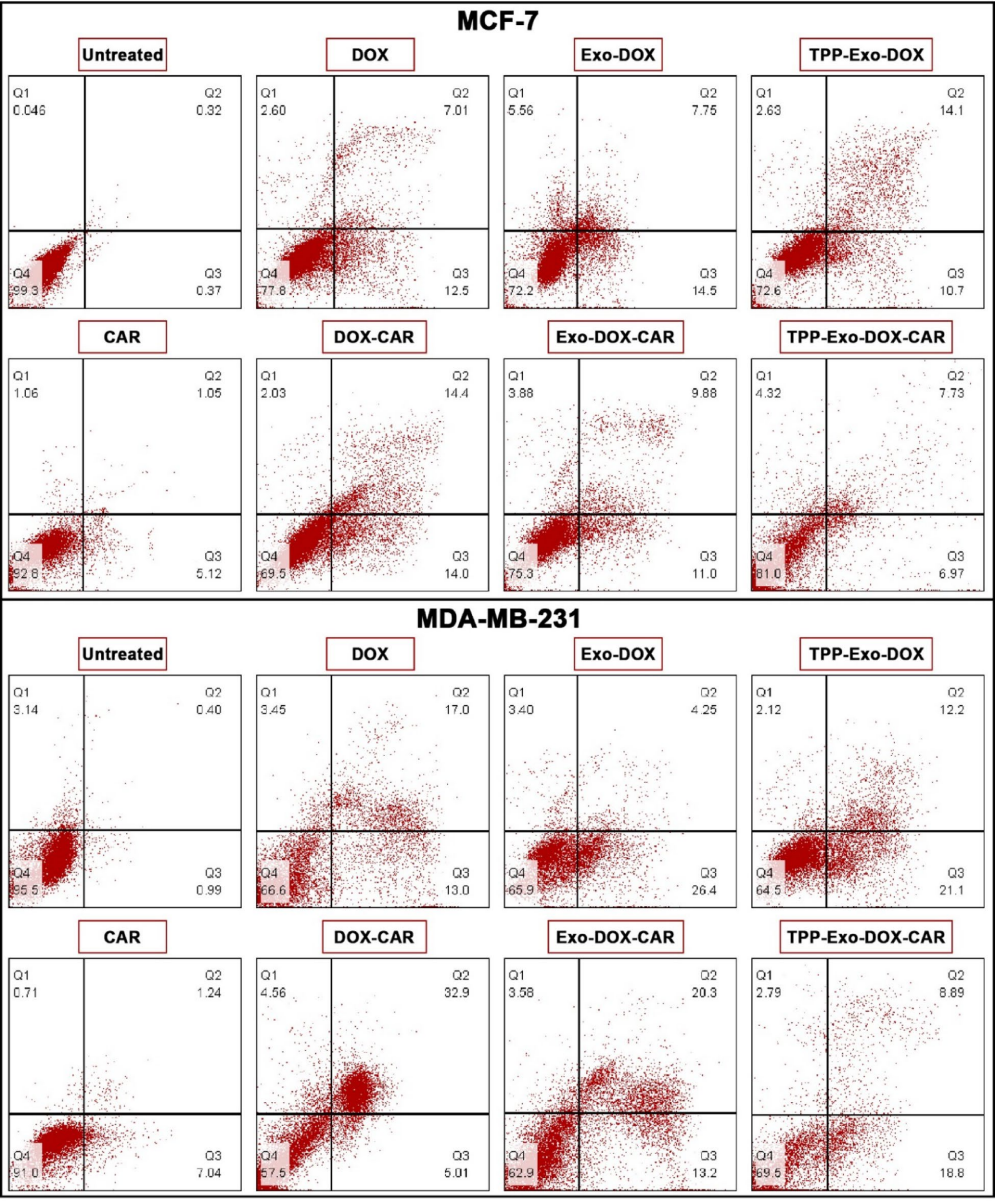
**Flow cytometric apoptosis analysis of MCF-7 and MDA-MB-231 cell after 48 h treatment** Representative flow cytometry dot plots showing Annexin V-FITC/PI staining of cell lines after treatment with DOX-CAR containing formulations. Quadrant analysis identifies viable cells (lower left), early apoptotic cells (lower right), late apoptotic cells (upper right), and necrotic cells (upper left).

Apoptotic effects of DOX-CAR formulations were assessed using Annexin V-FITC/PI analysis as depicted in Fig. 5. The comparison of apoptosis rate in formulations was shown in Supplementary Fig. S8. While CAR exhibited minimal toxicity (~ 6%) at this concentration (0.1 µM), its presence enhanced the apoptotic response of DOX-CAR and Exo-DOX-CAR comparing to DOX and Exo-DOX respectively. Studies show that increase in ROS levels could activate proapoptotic pathways^27^. Targeting DOX to mitochondria, whether through nanoparticles or TPP-conjugated DOX, enhances apoptosis rate^28,29^. The apoptosis assay confirmed that the cytotoxicity of DOX and DOX-CAR formulations was predominantly mediated through apoptotic pathways. Consistent with cellular ROS content and mitochondrial depolarization measurements, TPP-Exo-DOX-CAR showed reduced apoptosis compared to TPP-Exo-DOX in both cell lines.

## Inhibition of cell migration

**Fig. 6:**
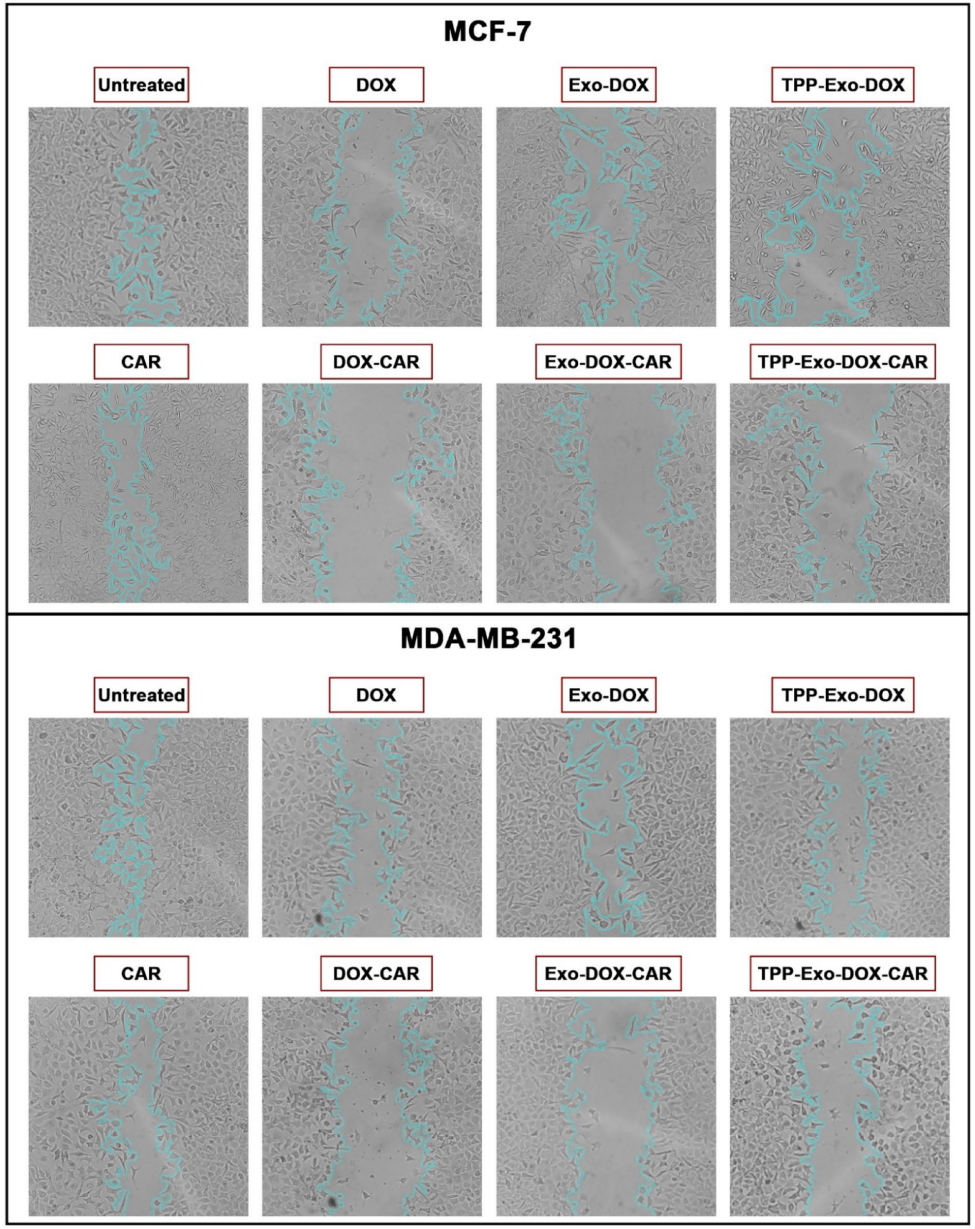
**Wound healing assay illustrates the migration potential of MCF-7 and MDA-MB-231 cells after 24 h treatment** Representative microscopic images (40x magnification) showing the wounded area 24 h after treatment with various formulations.

A wound healing assay was performed to evaluate the effects of CAR, DOX, and their combinations on migration potential. Figure 6 presents representative images of the two cell lines following 24 h treatment with DOX formulations at 0.1 µM concentration, both with 0.1 µM CAR in DOX-CAR formulations. The quantitative wound closure results are shown in Supplementary Fig. S9.

Compared to the untreated control group, CAR treatment significantly reduced wound closure rate. When administered alone, DOX exhibited a substantial inhibitory effect on cellular migration in both cell lines. Quantitative analysis showed that DOX treatment resulted in 28.6% and 31.2% wound closure percentages for MCF-7 and MDA-MB-231 cells, respectively.

The combination therapy revealed that DOX-CAR treatment substantially suppressed cellular migration in both lines. In comparison, treatment with DOX-CAR and Exo-DOX-CAR resulted in significantly lower wound closure rates: 10.7% and 14.1% in MCF-7 cells and 7.1% and 18.6% in MDA-MB-231 cells, respectively. Interestingly, while previous experimental assays had shown that TPP-Exo-DOX demonstrated significantly enhanced anticancer effects, in this migration assessment, both TPP-Exo-DOX and TPP-Exo-DOX-CAR exhibited comparable inhibitory effects on cellular migration, and CAR neither improved nor diminished the TPP-Exo-DOX efficacy.

## Discussion

Studies indicate that combination approaches using chemotherapy drugs alongside co-administered agents with anticancer activity can improve treatment effectiveness^30^. Drug repurposing represents a valuable strategy in cancer treatment development, offering reduced development costs and well-established safety profiles. In recent studies, CAR has documented cardioprotective effects against chemotherapy-induced toxicity and has shown anticancer properties^31,32^. The clinical use of CAR in preventing DOX-induced cardiotoxicity has led to investigations of their combined anticancer effects.

The Chou-Talalay analysis confirmed strong synergistic interactions at a 1:1 ratio in both MCF-7 and MDA-MB-231 cell lines. The combination effects of DOX and CAR were evaluated through multiple experimental approaches. The observed interactions revealed duality in CAR’s effects on mitochondrial function. While CAR is widely recognized for its cardioprotective properties, including mitochondrial protection and ROS reduction, the results showed that it could simultaneously enhance DOX-induced mitochondrial damage in cancer cells. This paradoxical effect was particularly evident in the increased ROS production and decreased MMP observed in DOX-CAR combinations compared to DOX alone.

The development of targeted delivery systems further illuminated the complexity of CAR and DOX interactions. While mitochondrial targeting through TPP conjugation enhanced DOX’s cytotoxicity (TPP-Exo-DOX), the same targeting strategy with the drug combination (TPP-Exo-DOX-CAR) showed reduced toxicity. This unexpected finding suggests that CAR may exert protective effects when co-delivered to mitochondria with DOX, as evidenced by reduced ROS generation and apoptosis rate. This in vitro observation indicates that mitochondrial co-delivery of CAR and DOX may potentially influence the therapeutic selectivity profile, though the underlying mechanisms require further investigation.

DOX treatment could increase oxidative stress through several mechanisms, with two primary mitochondria-related pathways significantly contributing to ROS production and cellular death. The first mechanism involves DOX’s affinity for cardiolipin, a critical phospholipid in the inner mitochondrial membrane, which disrupts mitochondrial structure and function by interfering with cytochrome c anchoring and compromising oxidative phosphorylation (OXPHOS). This interaction ultimately triggers increased ROS formation and apoptotic pathways^26,33^.

Second, DOX interacts with Complex I (NADH dehydrogenase) of the electron transport chain, where it undergoes a redox quinone cycle that generates an unstable semiquinone intermediate which rapidly auto-oxidizes by transferring electrons to molecular oxygen, establishing a continuous redox cycle that leads to progressive accumulation of superoxide and hydrogen peroxide within mitochondrial systems while severely compromising cellular OXPHOS capabilities and amplifying mitochondrial-induced oxidative damage^26,33^. The TPP-Exo-DOX demonstrated higher ROS content than Exo-DOX, but TPP-Exo-DOX-CAR showed reduced ROS content compared to TPP-Exo-DOX. Research using isolated rat organ mitochondria demonstrated that CAR significantly inhibits Complex I activity without apparent tissue selectivity^34^. This finding may explain the reduced cytotoxicity of TPP-Exo-DOX-CAR compared to TPP-Exo-DOX, as CAR can reduce ROS production by diminishing semiquinone formation through NADH dehydrogenase^35^.

Increased ROS production can cause mitochondrial dysfunction. MMP serves as a critical parameter for assessing mitochondrial function and cellular integrity. The results demonstrated that CAR induced a significant MMP disruption in both MCF-7 and MDA-MB-231 cell lines. Previous research has established that β-blockers like propranolol can modulate energy metabolism in triple-negative breast cancer^36^. Furthermore, analysis of mitochondrial respiration through oxygen consumption rate and glycolytic activity via extracellular acidification rate revealed that propranolol, nebivolol, and CAR substantially decreased mitochondrial respiratory functions in medulloblastoma cells, independent of their baseline bioenergetic status. The data indicated reduced basal and maximal respiration following 24 h treatment with these compounds at concentrations ranging from IC20 to IC80. ATP production was markedly diminished across all four medulloblastoma cell lines exposed to these β-blockers, even at minimal concentrations^37^.

Similar to DOX, imatinib demonstrates inhibitory effects on mitochondrial function. Previous research examining the combination of imatinib and CAR revealed that CAR induces severe mitochondrial damage in C6 rat glioma cell lines. TEM analysis of C6 glioma spheroids treated with CAR showed significant mitochondrial alterations, including swelling, cristae damage, and formation of myelin figures within the mitochondria. While CAR’s inhibitory effect on breast cancer cell lines was anticipated based on these findings, it is noteworthy that numerous studies have reported CAR’s mitochondrial protective properties, including inhibition of ROS production, free iron chelation, maintenance of mitochondrial membrane potential, and preservation of cellular energy homeostasis^3,4,38^. Therefore, CAR’s potential prevention of DOX induced mitochondrial damage could have been reasonably expected, which has otherwise been observed in both the C6 treatment and the current study.

Mitochondrial targeting of DOX resulted in further depolarization of MMP, correlating with increased ROS production. However, TPP-Exo-DOX-CAR demonstrated improved MMP compared to TPP-Exo-DOX. Investigations of CAR’s interactions with the OXPHOS system in isolated rat organ mitochondria have demonstrated that CAR significantly inhibits Complex I activity without tissue specificity. This inhibition was particularly evident under both phosphorylating and uncoupled conditions. Although CAR possesses inherent ROS scavenging properties, researchers discovered that its inhibitory effect on Complex I paradoxically increases ROS production. The study concluded that CAR’s pro-oxidant effects, resulting from respiratory chain inhibition, may supersede its intrinsic antioxidant properties^34^. These findings were subsequently validated in a study using H9c2 cardiomyoblast cell lines, which demonstrated a consistent decrease in mitochondrial Complex I activity alongside increased mitochondrial H₂O₂ production, accompanied by changes in total glutathione and protein thiols content^39^. These observations suggest that CAR could synergistically enhance ROS production with DOX in mitochondria. However, the results indicated that mitochondrial targeting of CAR activates its protective effects, and as observed, CAR in TPP-Exo-DOX-CAR formulation showed improved outcomes.

As a beta-blocker, CAR could inhibit invasion and migration, as previously demonstrated for propranolol. The modulation of multiple signaling pathways is thought to be responsible for these effects^40,41^. Both DOX-CAR and Exo-DOX-CAR displayed more potent inhibition of cellular proliferation and invasion compared to DOX or Exo-DOX treatments. These findings strongly indicate the synergistic efficacy of DOX-CAR in targeting breast cancer cells, suggesting its potential as a therapeutic agent.

Migration studies revealed another dimension of CAR’s anticancer potential, demonstrating significant inhibition of cell migration independent of its interaction with DOX. The DOX-CAR combination therapy showed enhanced anti-migration effects compared to either drug alone, indicating multiple complementary effects.

These findings underline the critical importance of comprehensive in vitro studies in understanding the DOX and CAR interactions, particularly for repurposed drugs like CAR. The results showed that CAR synergistically enhances DOX toxicity in both tested cell lines by increasing ROS production, inducing MMP depolarization, and consequently promoting apoptosis. In contrast, mitochondrial targeting of the DOX-CAR combination using TPP-Exo-DOX-CAR reduces toxicity compared to TPP-Exo-DOX by decreasing ROS production and the rate of apoptosis.

Future research should focus on elucidating the precise mechanisms governing CAR’s differential effects in normal versus cancer cells, particularly in the context of mitochondrial function. Additionally, in vivo studies will be crucial to validate these findings and assess the potential clinical applications of various delivery systems developed in this study.

## Materials and methods

### Materials

CAR was generously provided by Hakim Pharmaceutical Company. Doxorubicin hydrochloride (DOX) was obtained from Hetero Healthcare LTD (India). Cell culture essentials, including Dulbecco’s Modified Eagle Medium (DMEM), penicillin/streptomycin solution, and phosphate-buffered saline (PBS), were purchased from BioIdea Inc. (Iran). Fetal bovine serum (FBS) was procured from Biowest (France).

Chemical reagents were primarily acquired from Sigma-Aldrich (USA), including: (4-carboxybutyl) triphenylphosphonium bromide (TPP), N-(3-Dimethylaminopropyl)-N’-ethylcarbodiimide hydrochloride (EDC), N-Hydroxysuccinimide (NHS), 3-(4,5-dimethylthiazol-2-yl)−2,5-diphenyltetrazolium bromide (MTT), 2’,7’-dichlorodihydrofluorescein diacetate (DCFDA), rhodamine 123 (Rh123), DAPI, and Annexin V-FITC/PI apoptosis detection kit. Dimethyl sulfoxide (DMSO) of analytical grade was acquired from Merck (Germany). Antibodies anti-CD63-FITC and anti-CD9-APC were purchased from BD Biosciences (San Jose, CA, USA). The exosome isolation kit (Exocib) and Bradford protein assay kit (Protocib) were obtained from Cibbiotech (Iran). Unless explicitly stated otherwise, all additional chemicals and reagents were of analytical grade and sourced from Merck.

### Cell culture

MCF-7 and MDA-MB-231 human breast cancer cell lines were acquired from the National Cell Bank of Iran, Pasteur Institute (Tehran, Iran). Cells were maintained in high-glucose DMEM supplemented with 10% fetal bovine serum and 100 U/mL penicillin/streptomycin, and incubated in a humidified environment with 5% CO2 at 37 °C.

### In vitro cytotoxicity assay

For MTT viability assays, cells were plated at a density of 2 × 10^3^ cells per well in 96-well plates. Following a 24-hour incubation period, cells were exposed to varying treatment concentrations and incubation continued for another 48 h. Post-treatment, the media was discarded, and 100 µL of MTT solution (0.5 mg/mL) was added to each well and incubated for 4 h. Resulting formazan crystals were solubilized in 100 µL DMSO, and absorbance was determined at 545 nm using a Stat Fax 3200 spectrophotometer (Awareness Technology, USA). Cell viability was calculated as a percentage relative to the untreated control group.

### Analysis of drug combination effects

The interaction between CAR and DOX were investigated using the median effect principle established by Chou and Talalay^42^. The MTT assay results were transformed into a fraction affected (Fa) scale, ranging from 0 to 1, representing cell viability from 100% to 0%, respectively. Utilizing CompuSyn software (Version 1.0), combination index (CI) and drug reduction index (DRI) were computed. The CI values were interpreted as follows: CI < 1 indicated synergistic effects, CI = 1 represented additive interactions, and CI > 1 suggested antagonistic drug interactions^43^.

### Exosome isolation and characterization

To isolate exosomes, cells underwent an adaptation process to serum-free medium, strategically designed to minimize contamination from fetal bovine serum (FBS)-derived exosomes. Initially, cells were cultured to achieve approximately 60% confluency in complete medium. Subsequently, the medium was progressively replaced with serum-free media by decreasing the FBS content by 3% on a daily basis to reach media with less than 1% FBS after 3 days^44^.

After a 48-hour conditioning period, exosomes were extracted from the conditioned media using the Exocib exosome extraction kit. Initially, cellular debris was eliminated through centrifugation at 4000 g for 10 min, followed by filtration using a 0.22-µm syringe filter to remove larger particulate matter. The subsequent exosome isolation proceeded according to the manufacturer’s protocol. Isolated exosome aliquots were preserved at − 70 °C for future experimental applications.

Comprehensive characterization of the isolated exosomes was performed using multiple analytical techniques. The size distribution and zeta potential were analyzed through dynamic light scattering (DLS) employing the NANO-flex apparatus (Particle Metrix, Germany) and Zetasizer Nano ZS instrument (Malvern Instruments Ltd., UK), respectively. Total protein content was quantified using the Bradford assay kit. Exosome purity was assessed by evaluating the exosomal markers CD63 and CD9 through flow cytometry using the FACSCalibur (BD Biosciences, USA).

Transmission electron microscopy (TEM) was employed to visualize exosomes morphology. Purified exosomes underwent negative staining with 2% uranyl acetate for 1 min at room temperature. After removing excess stain, the samples were examined using a transmission electron microscope (EM208S; Philips, Netherlands).

### Drug loading

100 µL of exosome suspension (1 mg/mL protein concentration) were sonicated with DOX and CAR at molar ratios of 2:1 and 1:1, maintaining a total drug amount of 100 µg. Sonication was performed in an ice-cold bath using parameters of 100 W, 20 kHz, 20% power, with a 1 s on/off pulse cycle. The procedure comprised 6 cycles of 30 s on/off periods, with a 2 min ice incubation between every two cycles, followed by a 60 min incubation at 37 °C after the final cycle. To remove unincorporated free drugs, the sample underwent ultracentrifugation at 60,000 × g for 40 min at 4 °C^45^. The resulting supernatant was analyzed for residual drug concentration using UV-Vis spectroscopy (Varian-CARY50), with absorbance measurements performed at 480 nm for DOX and 245 nm for CAR.

Drug loading capacity was calculated using the following equation, where Dt represents the total drug added and Ds represents the drug remaining in the supernatant:

Loading capacity = (Dt - Ds)/Exo (Exosomal protein weight) × 100.

### *Invitro* drug release study

The in vitro release profiles of drugs were analyzed using the dialysis method under two different pH conditions simulating physiological and endo-lysosomal environments (PBS pH 7.4 and acetate buffer pH 5.5, respectively)^46^. Both buffers contained 1% Tween 80 to maintain sink conditions. The Exo-DOX-CAR nanovesicle was prepared at a protein concentration of 1.0 mg/mL in each release buffer. 1 mL of the nanovesicle suspension was transferred into a dialysis bag (MWCO 12 kDa) and immersed in 5 mL of the respective buffer medium. The setup was maintained at 37 °C with continuous gentle shaking at 200 rpm. At predetermined time points (0, 1, 3, 6, 12, 24, and 48 h), 1 mL of release medium was withdrawn and replaced with an equal volume of fresh buffer. The concentration of released drug was determined using UV-Vis spectrophotometry.

### Functionalization of exosomes with TPP

Surface functionalization of Exos for mitochondria-targeted delivery was performed through covalent attachment of the carboxyl group of TPP with the amine groups present on the Exo surface using the EDC-NHS coupling method. To activate the TPP carboxyl groups, 200 mg of TPP was combined with EDC (70 mg) and NHS (55 mg) in 2 ml PBS, and the mixture was allowed to react for 1 h. 1 ml of Exo-DOX or Exo-DOX-CAR suspension (200 mg/ml) in PBS was added to the activated TPP, the NHS ester rapidly reacted with the surface amino groups^47^. The mixture was then incubated for 30 min at room temperature with gentle stirring to ensure uniform conjugation. To remove non-conjugated TPP, the reaction mixture underwent ultracentrifugation for 40 min at 60,000 g at 4 °C. Following ultracentrifugation, the sample was washed with PBS to further eliminate unbound molecules. The successful TPP conjugation was subsequently assessed by measuring the zeta potential, as described in previous step.

### *In vitro* cellular uptake

To investigate the uptake and mitochondrial targeting ability of TPP-Exos, DOX containing formulations were incubated with their Exo-originating cells. MCF-7 and MDA-MB-231 cells were seeded in 48-well plates at a density of 5 × 10^3^ cells/well and incubated at 37 °C for 24 h. The cells were then treated with free DOX, Exo-DOX, or TPP-Exo-DOX, containing equivalent amounts of DOX (5 µM) for 8 h. After removing the culture medium, cells were treated with 200 nM MitoTracker Green for 1 h. The intracellular localization was tracked using a Cytation 3 Cell Imaging Multi-Mode Reader (BioTek Instruments, USA)^48^.

### Measurement of reactive oxygen species

Intracellular ROS generation was determined using DCFDA, a cell-permeable non-fluorescent probe that is cleaved by cellular esterases and subsequently oxidized by ROS to form the fluorescent compound 2’,7’-dichlorofluorescein (DCF). Cells were seeded in 48-well plates at a density of 5 × 10^4^ cells/well and incubated for 24 h to allow attachment. The cells were then treated with free DOX, free CAR, Exo-DOX, TPP-Exo-DOX, Exo-DOX-CAR, and TPP-Exo-DOX-CAR formulations for 24 h, with DOX concentration maintained at 0.1 µM across all treatments. Following treatment, cells were washed with PBS and incubated with FBS-free medium containing 10 µM DCFDA for 45 min at 37 °C in a 5% CO₂ incubator. The fluorescence intensity was measured using a Cytation 3 Cell Imaging Multi-Mode Reader (BioTek Instruments, USA) at excitation and emission wavelengths of 485 nm and 530 nm, respectively^49^. To address potential fluorescence interference from DOX, each treatment group containing DOX was run in six replicates. After the incubation period, three replicates were stained with DCFDA, while the other three replicates were left unstained and used as internal controls. The fluorescence intensity from DOX was subtracted from the total signal in stained samples to ensure accurate quantification and eliminate potential artifacts.

For ROS content assessment by flow cytometry, cells were seeded in a 12-well plate at a density of 2 × 10⁵ cells/well and incubated for 24 h. The treatments described above were applied, and cells were incubated for an additional 24 h. For ROS detection, cells were incubated with DCFDA solution for 45 min at 37 °C in the dark. Following staining, cells were gently trypsinized, collected, and resuspended in cold PBS. Samples were immediately evaluated by flow cytometry to quantify DCF fluorescence intensity. The data were analyzed using FlowJo software (version 10.5.3, BD Life Sciences, Ashland, OR, USA; https://www.flowjo.com).

### Mitochondrial membrane potential assay by Rhodamine 123

Changes in MMP were evaluated using Rh123, a fluorescent dye that accumulates in mitochondria based on membrane potential. Rh123 selectively accumulates in mitochondria with intact, high membrane potentials, and its accumulation decreases upon mitochondrial depolarization. Cells were seeded in 48-well plates at a density of 5 × 10^4^ cells/well and incubated for 24. Following incubation, cells were treated with free DOX, free CAR, DOX-CAR, Exo-DOX, TPP-Exo-DOX, Exo-DOX-CAR, and TPP-Exo-DOX-CAR for 24 h, with a DOX concentration of 0.1 µM across all treatments. After treatment, the culture medium was aspirated, and cells were washed twice with PBS. Cells were then incubated with 10 µM Rh123 in serum-free medium for 30 min at 37 °C in the dark. The fluorescence intensity was measured using a Cytation 3 Cell Imaging Multi-Mode Reader (BioTek Instruments, USA) at excitation and emission wavelengths of 490 nm and 522 nm, respectively^50^. Each DOX-containing group was run in six replicates. Three replicates remained unstained as internal control groups. The fluorescence intensity from DOX was subtracted from the total signal in stained samples to eliminate DOX signal interference.

### Determination of apoptosis

Cell death mechanisms were investigated through dual labeling with Annexin V-FITC/PI. MCF-7 and MDA-MB-231 were cultured in 6-well plates (2 × 10⁵ cells per well) for 24 h prior to treatment. The cells were exposed to various formulations including free DOX, free CAR, Exo-DOX, TPP-Exo-DOX, Exo-DOX-CAR, and TPP-Exo-DOX-CAR, with DOX maintained at 0.1 µM in all treatment groups for 48 h. Following the manufacturer’s protocol for the apoptosis detection kit, cells were stained with Annexin V-FITC and PI. Flow cytometric analysis was performed on a BD FACSCalibur system (BD Biosciences), evaluating a minimum of 10,000 events per sample. The cell populations were categorized using FlowJo software (version 10.5.3, BD Life Sciences, Ashland, OR, USA) into four quadrants: viable cells (FITC^−^/PI^−^), early apoptotic cells (FITC^+^/PI^-^), late apoptotic cells (FITC^+^/PI^+^), and necrotic cells (FITC^−^/PI^+^).

### Cell migration assay

Cell migration capabilities of MCF-7 and MDA-MB-231 cell lines were evaluated using a wound healing assay. Cells were plated in 48 well plates at a density of 7 × 10^4^ cells per well and allowed to grow until reaching 90% confluency. A linear scratch was carefully created across the cell monolayer using a sterile 100 µL pipette tip. Following the scratch formation, the wells were rinsed twice with PBS to eliminate detached cells. The cells were subjected to different treatments and observed after 24 h. Microscopic images were captured at the initial timepoint (0 h) and 24 h after treatment using an inverted microscope. Migration rates were calculated using the formula: Migration rate (%) = (initial wound width - final wound width)/initial wound width × 100%. Quantification of scratch areas was performed using ImageJ software^51^.

### Statistical analysis

Statistical analyses were conducted using version 10.4 of GraphPad Prism (GraphPad Software, San Diego, CA, USA). Student’s t-tests were utilized to compare two experimental groups. For comparisons of more than two groups, one-way ANOVA was used, followed by Tukey’s post hoc analysis. For experiments involving multiple variables, two-way ANOVA with Sidak’s post hoc test was implemented. Each experiment was performed in triplicate, and data are shown as mean ± SD. Statistical significance is indicated by: *P < 0.05, **P < 0.01, ***P < 0.001, ****P < 0.0001.

## Supplementary Information

Below is the link to the electronic supplementary material.

Supplementary Material 1

## Data Availability

All data generated or analyzed during this study are included in this published article (and its Supplementary Information files).
