# Supplementary material for: Modified exosomes for targeted delivery of doxorubicin and carvedilol to mitochondria in breast cancer cells
Source: Sci Rep. 2025 Nov 3;15:38386. doi: 10.1038/s41598-025-22156-2 (PMC12583512; doi:10.1038/s41598-025-22156-2)
Supplement: Supplementary file 1 — Supplementary Material 1 [file 41598_2025_22156_MOESM1_ESM.docx]

**Modified exosomes for targeted delivery of doxorubicin and carvedilol to mitochondria in breast cancer cells**

Tooba Soudi^1^, Fatemeh Bagheri^1*^, Seyed Abbas Shojaosadati^1^, Mohsen Rezaei^2^, Ehsan Motamedian^1^

^1^Department of Biotechnology, Faculty of Chemical Engineering, Tarbiat Modares University, Tehran, Iran

^2^Department of Toxicology, Faculty of Medical Sciences, Tarbiat Modares University, Tehran, Iran

Corresponding author: Fatemeh Bagheri

E-mail address: [f.bagheri@modares.ac.ir](mailto:f.bagheri@modares.ac.ir)

Postal address: Tarbiat Modares University, Jalal Ale Ahmad Street, P.O.Box: 14115-111, Tehran, Iran. Fax number: +982182884931


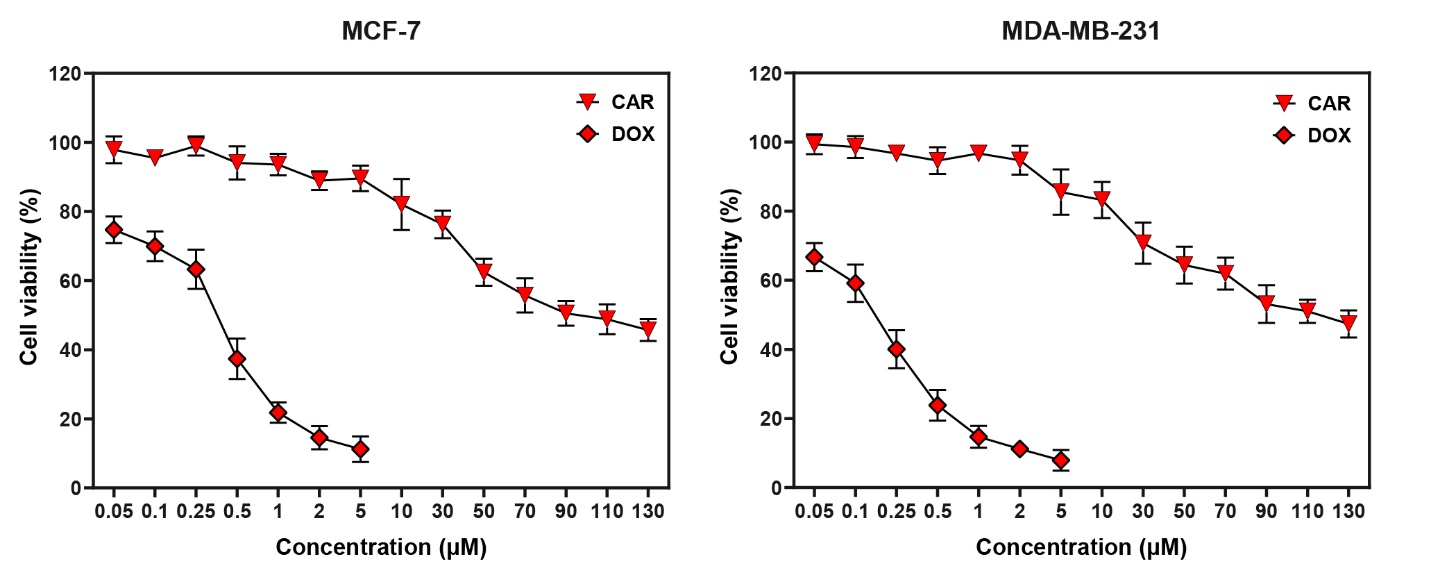


**Fig. S1 Cell viability of DOX and CAR on MCF-7 and MDA-MB-231 cell lines** The viability of MCF-7 and MDA-MB-231 cell lines assessed by MTT assay after 48 h treatment with DOX and CAR. Data are presented as mean ± SD of three replicates


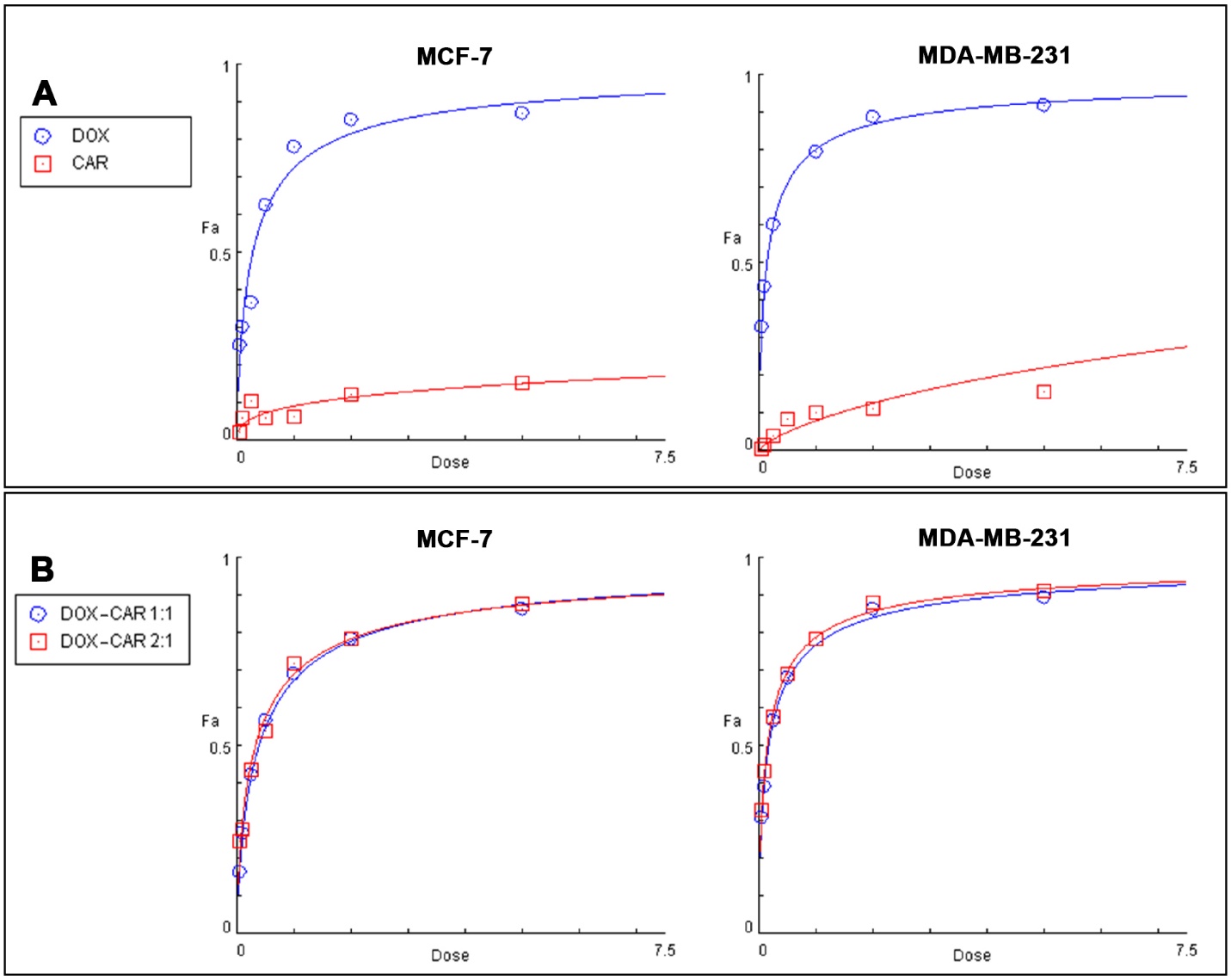


**Fig. S2 Dose-response curves for single and combination treatment** The Fa-Dose plot illustrates the cytotoxicity of DOX and CAR as single agents (A), and in combination by DOX to CAR ratio of 1:1 and 2:1 ratio respectively (B) in MCF-7 and MDA-MB-231 cell lines. The x-axis represents drug concentrations in µM and the Fa values are the average of three replicates


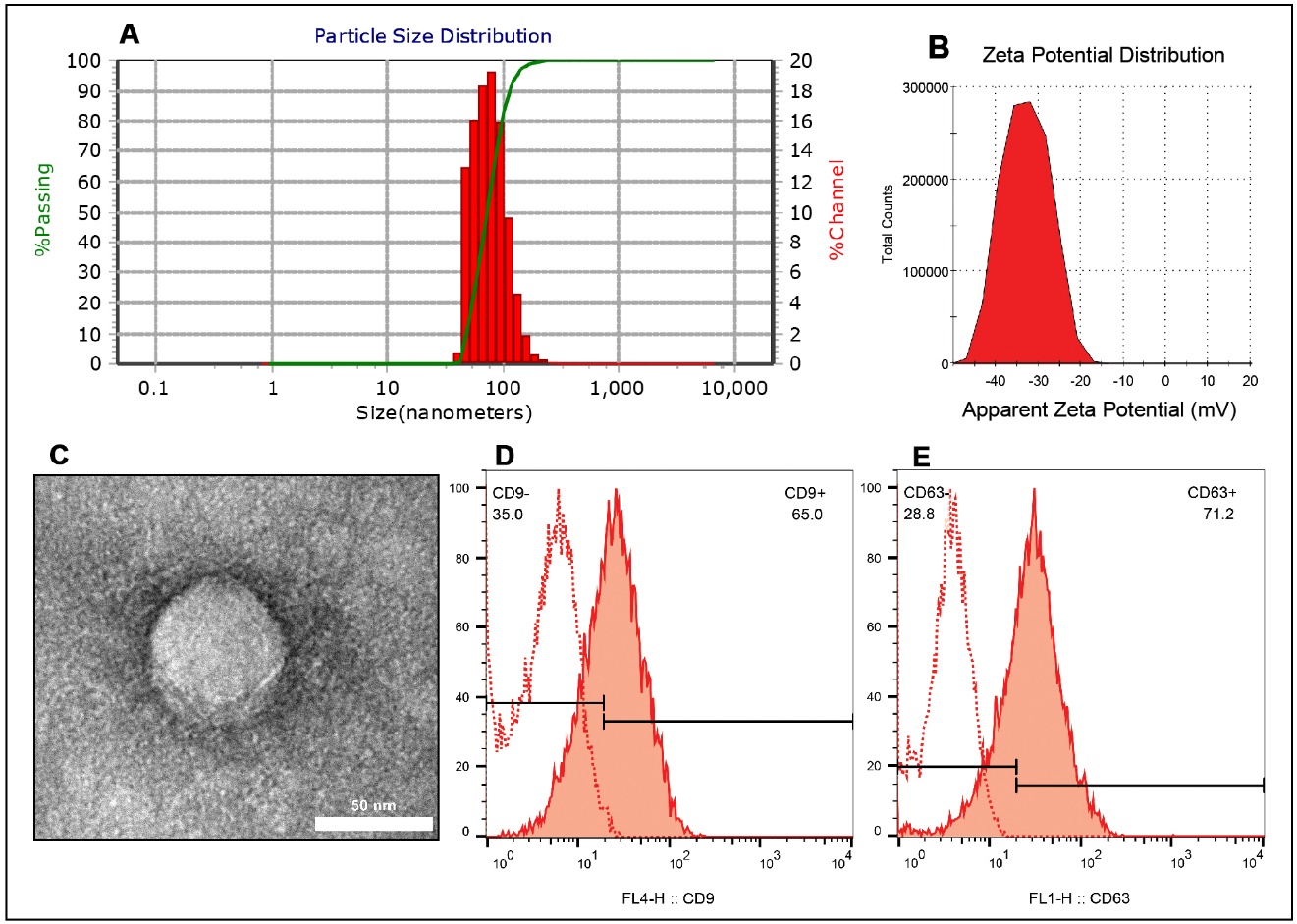


**Fig. S3 Characterization of Exo derived from MCF-7 condition media** (A) Size distribution profile of isolated Exos, (B) Surface charge characterization by zeta potential analysis, (C) TEM image of negatively stained Exos, Flowcytometric analysis of Exo surface markers CD9 (D) and CD63 (E)


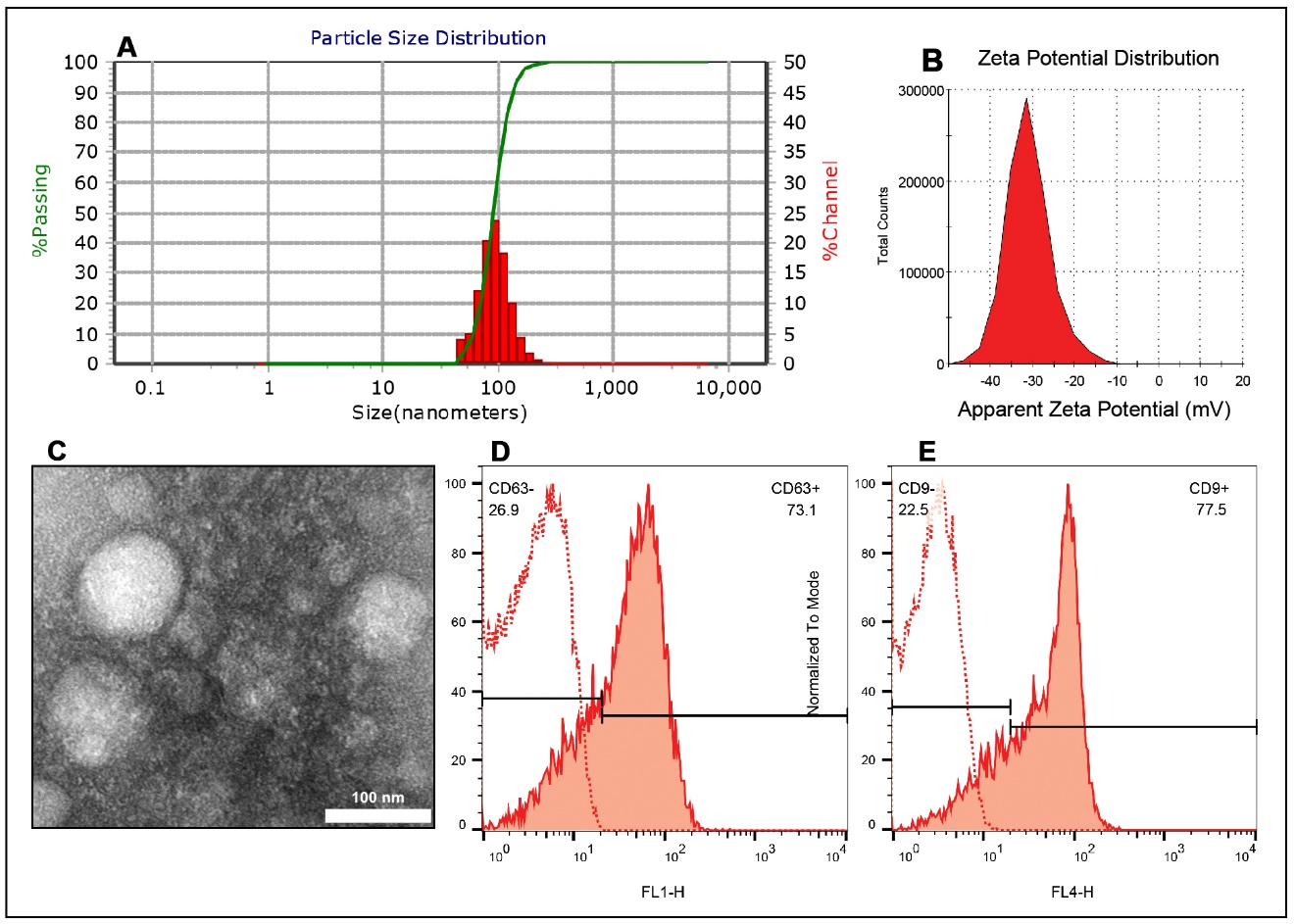


**Fig. S4 Characterization of Exo derived from MDA-MB-231 conditioned media** (A) Size distribution profile, (B) Surface charge characterization by zeta potential analysis, (C) TEM image of negatively stained Exos, Flowcytometric analysis of Exo's surface markers CD9 (D) and CD63 (E)


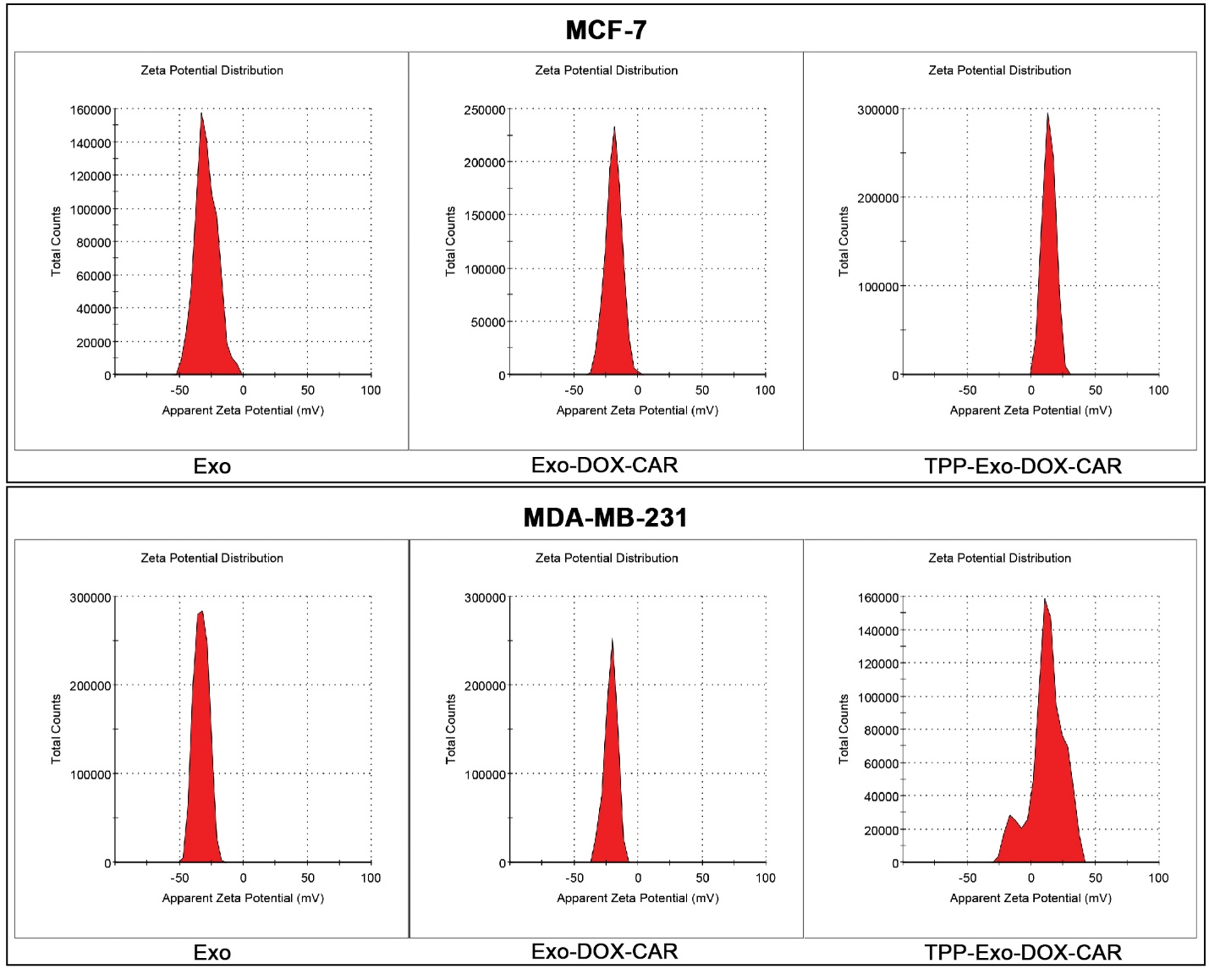


**Fig. S5 Zeta potential analysis of exosomal formulations** Zeta potential measurements of Exo, Exo-DOX-CAR, and TPP-Exo-DOX-CAR. The positive shift in zeta potential confirms successful TPP conjugation


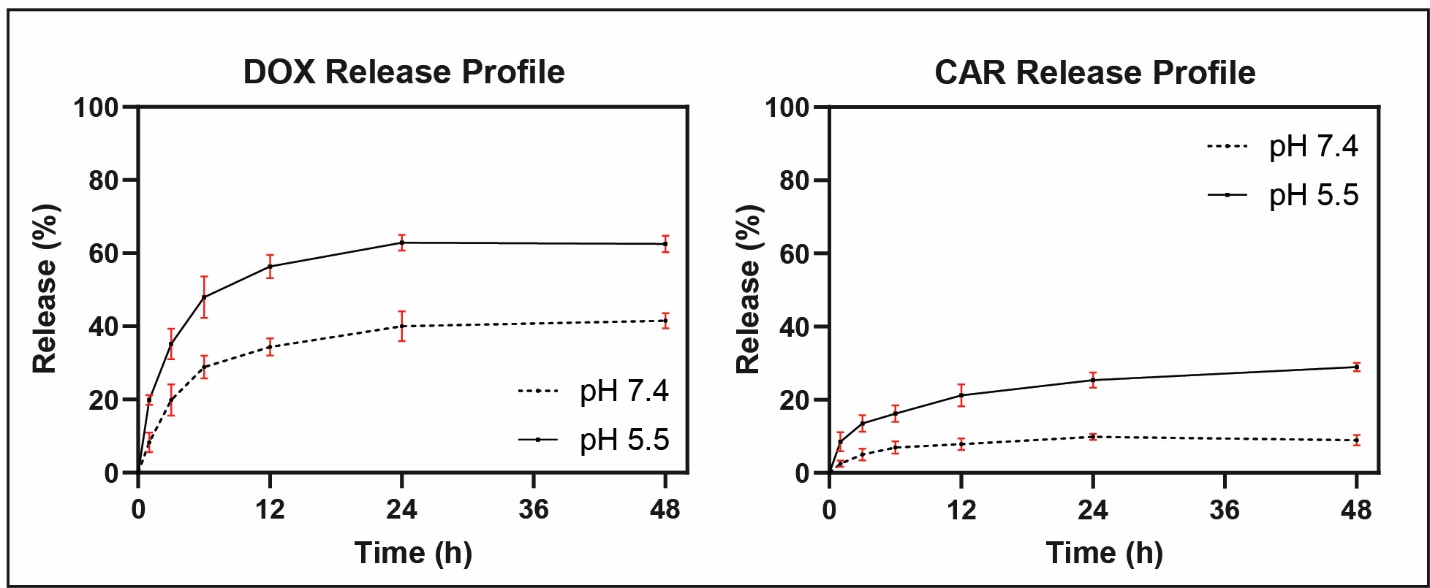


**Fig. S6 Release profile of DOX and CAR from Exo-DOX-CAR** Cumulative drug release from Exo-drug formulations over 48 hours in buffer solutions at pH 7.4 and pH 5.5. Enhanced drug release was observed under acidic conditions (pH 5.5) compared to physiological pH (7.4)


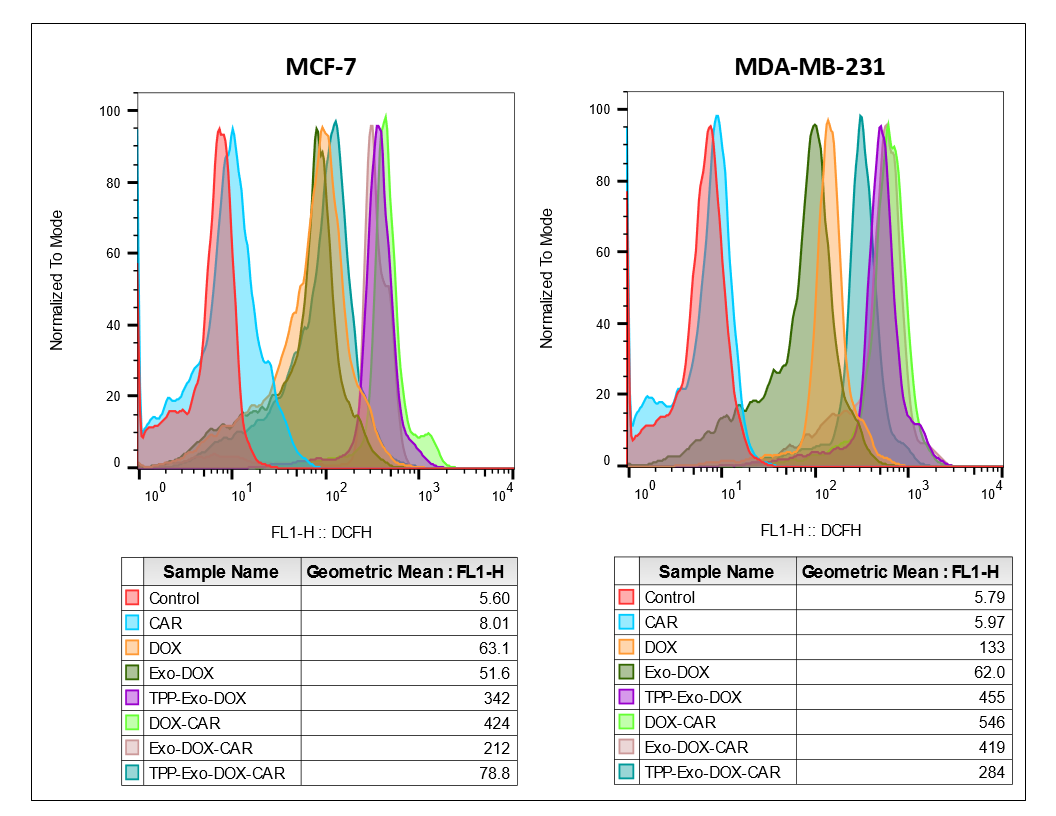


**Fig. S7 ROS content analysis by flow cytometry in MCF-7 and MDA-MB-231 cell lines** Cells were treated with DOX, CAR (0.1 µM), DOX-CAR, Exo-DOX, TPP-Exo-DOX, Exo-DOX-CAR, and TPP-Exo-DOX-CAR at an equivalent DOX concentration of 0.1 µM for 24 h, followed by DCFDA staining and flow cytometric analysis. DCF fluorescence intensity represents intracellular ROS levels.


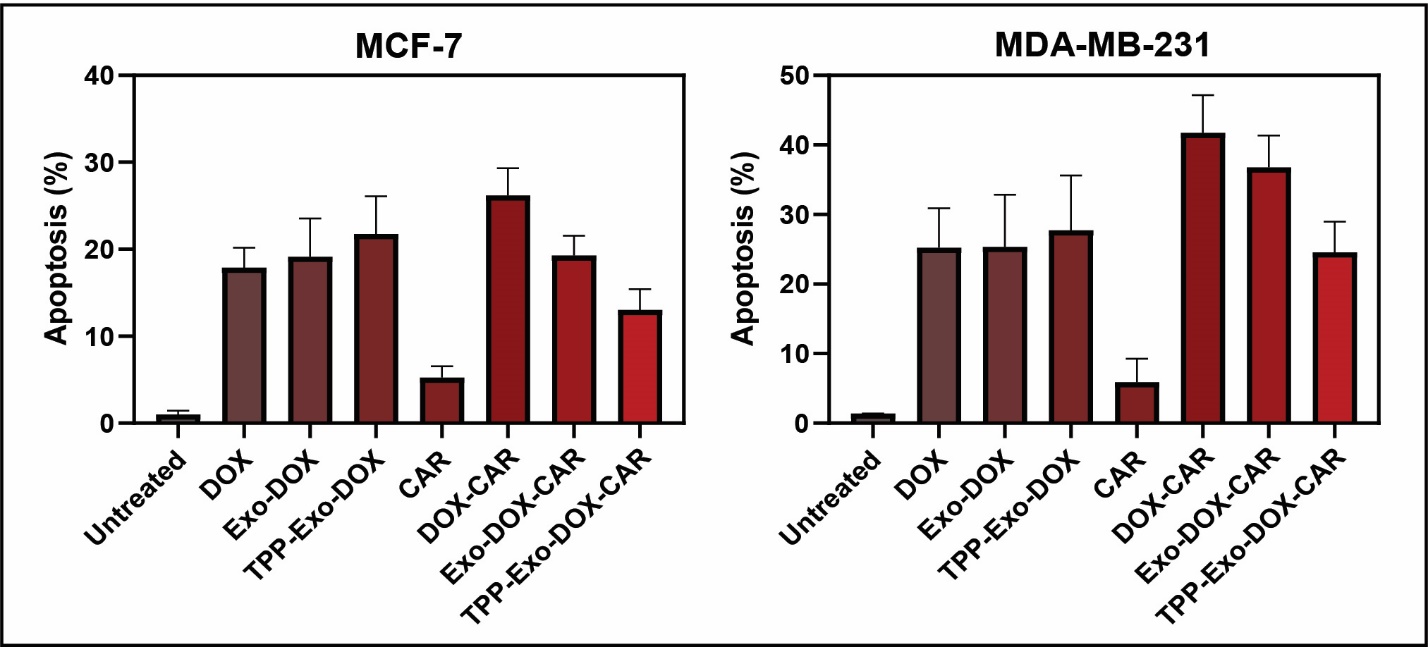


**Fig. S8 Comparison of apoptotic cell death after 48 h treatment of MCF-7 and MDA-MB-231 cells.** The percentage of apoptotic cells (early and late apoptosis combined) following 48 h treatment. TPP-Exo-DOX-CAR formulation induces lower levels of apoptosis compared to free DOX-CAR and Exo-DOX-CAR treatments


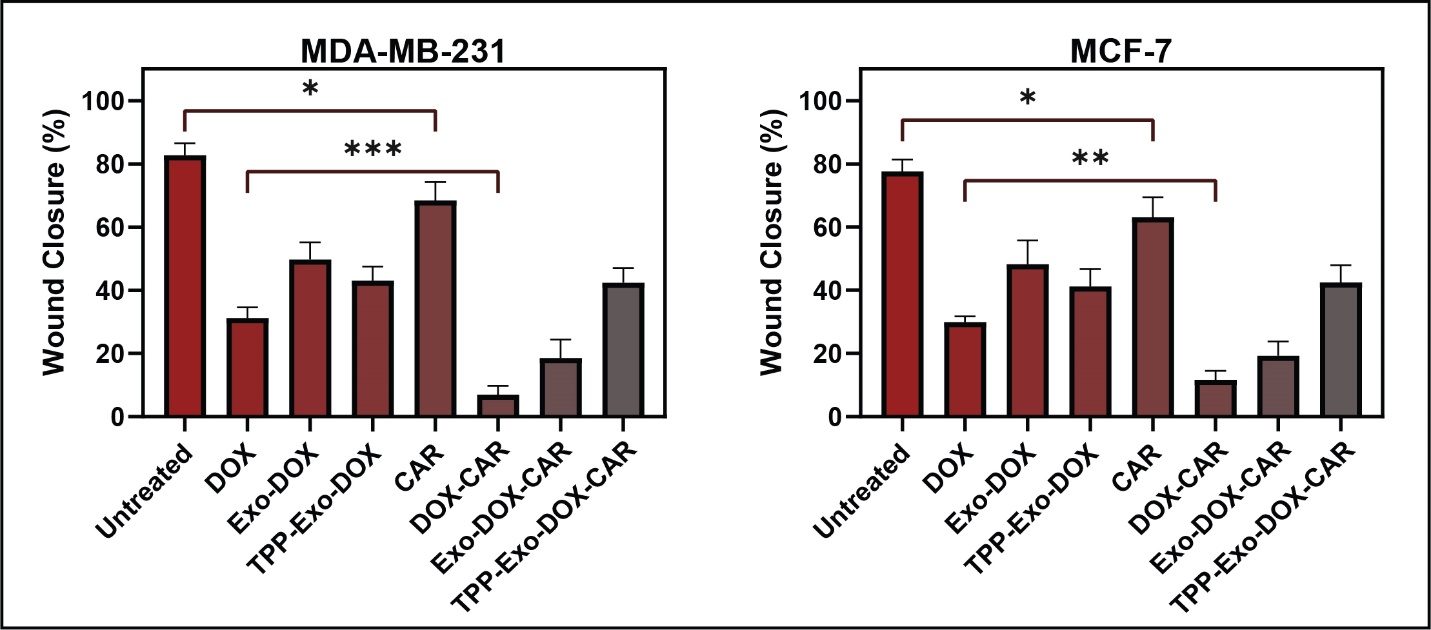


**Fig. S9 Wound closure quantitative analysis of MCF-7 and MDA-MB-231 cells after 24 h treatment** The percentage of wound closure compared to time 0 after 24h treatment. CAR reduced cell migration in all formulations, demonstrating its anti-migratory effect even at non-toxic concentrations
